# Chemical Crosslinking of Acid Soluble Collagen Fibres

**DOI:** 10.3390/biomimetics10100701

**Published:** 2025-10-15

**Authors:** Peter Schyra, Dilbar Aibibu, Bernd Sundag, Chokri Cherif

**Affiliations:** 1 Institute of Textile Machinery and High Performance Material Technology, TUD Dresden University of Technology, 01069 Dresden, Germany; 2 GfN Herstellung von Naturextrakten GmbH, 69483 Wald-Michelbach, Germany

**Keywords:** collagen, acid-soluble collagen, wet spinning, fibre, crosslinking, cytocompatibility

## Abstract

Collagen, as the predominant structural protein in vertebrates, represents a promising biomimetic material for scaffold development. Fibre-based scaffolds produced through textile technologies enable precise modulation of structural characteristics to closely mimic the extracellular matrix architecture using wet-spun collagen fibres. However, this in vitro fibre formation lacks natural crosslinking, resulting in collagen fibres with compromised mechanical strength, enzymatic resistance, and thermal stability compared to their native counterparts, thus restricting their biomedical applicability. Post-fabrication crosslinking is therefore imperative to enhance the durability and functional performance of collagen fibre-based scaffolds. Although traditional crosslinkers like glutaraldehyde effectively improve mechanical strength and stability, their clinical utility is hindered by cytotoxicity and associated adverse biological responses. Alternative synthetic crosslinking agents, such as hexamethylene diisocyanate, 1-Ethyl-3-(3’-dimethyl amino propyl) carbodiimide, and 1,4-Butanediol diglycidyl ether, have demonstrated superior cytocompatibility while effectively improving collagen fibre properties. Nonetheless, synthetic compounds may induce more pronounced foreign body reaction than natural agents, necessitating further investigation into their cytocompatibility across varying concentrations. In contrast, plant-based crosslinking offers a promising, cytocompatible alternative, significantly enhancing the thermal and mechanical stability of collagen fibres, provided that potential fibre discolouration is acceptable for intended biomedical applications.

## 1. Introduction

**Figure 1:**
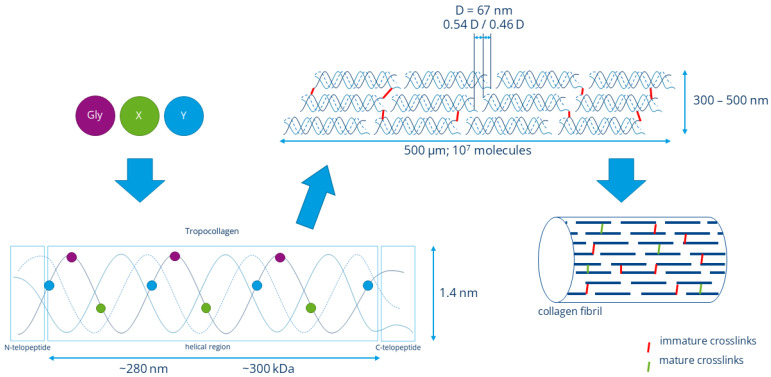
Basic structure of the fibrillar collagen type I.

Collagen is the most abundant protein in vertebrates, and of the 28 known types, type I collagen is its most frequent form found in most connective tissues like bone, skin, tendons, and ligaments [1,2,3]. Thanks to its favourable properties such as low antigenicity and (cross-species) immunogenicity [4], high tensile stress [5,6], and biodegradability [1], it is an established biomaterial for medical and cosmetic applications and has long served as a central model material in the field of biomimetics due to its hierarchical organisation, cytocompatibility, and mechanical performance. Especially in the field of tissue engineering and regenerative medicine, collagen is a suitable candidate for biomimetic scaffold fabrication and offers a versatile platform for fabricating fibrous scaffolds that emulate native tissue architecture. Therefore, a clear understanding of the underlying structure and properties of collagen is crucial to mimic the extracellular matrix (ECM) and mechanical behaviour of the target tissue. The basic quaternary structure of the fibrillary collagen type I is well investigated and given schematically in Figure 1. Starting from a primary structure of the amino acid sequence of [Gly-X-Y]_n_, collagen builds three left-handed polypeptide helical αchains arranged in a right-handed triple helix. The molecules containing these triple helices, called tropocollagen, can hierarchically arrange in form of micro fibrils (Ø20–40 nm) and fibrils (Ø300–500 nm). These fibrils arrange in bundles to form fibres (Ø4–12 µm) in vivo. The collagen fibres transduce forces, dissipate energy, and play a key role in the control of structural integrity, biochemical properties, and physiological functions of the ECM [7,8]. Another important aspect is the D-periodical banding of collagen fibrils. It is their main structural characteristic in the longitudinal direction and plays an important role in the cell signalling properties of collagen, as it organises collagen binding sites on the fibril surface in a regular order. In this way, D-periodicity influences cell adhesion, elongation, and motion directionality [9]. It is therefore evident that the design of biomimetic materials for tissue engineering and regenerative medicine, with the objective of supporting physiological cell functioning, requires the remodelling of the native ECM, which features a fine structure and nanotopography, including D-periodicity. To replicate these delicate ECM structures of body tissues for tissue substitutes, it is rational to use fibre-based scaffolds and textile–technological techniques. The use of collagen in fibre-based form is essential for the fabrication of biomimetic scaffolds, as it closely replicates the natural architecture of ECM. Fibre-based collagen structures provide a high specific surface area, which promotes cell adhesion, proliferation, and differentiation. Additionally, they enable the formation of porous 3D networks that are critical for cell migration, nutrient diffusion, and vascularisation. The fibrous form also imparts mechanical stability and elasticity to the scaffold, making it particularly suitable for applications in tissue regeneration. The textile fabrication techniques allow for precise control of fibre orientation and porosity, further enhancing the biomimetic properties of the material [10].

The basis for textile processing is the production of reconstituted collagen fibres. Several spinning procedures have been reported in the literature, featuring discontinuous [11,12,13,14,15] or continuous [10,16,17,18,19] fibre production. The spinning of these collagen fibres is based on collagen’s inherent ability to self-assemble in fibrils under optimal conditions [20,21,22]. The first developed spinning procedures used a top-down approach, producing dispersions of insoluble collagen. These dispersions contain small fractions of fully developed collagen fibrils, including D-periodic banding and natural crosslinks [23,24,25]. But the natural fibres are randomly disrupted during fragmentation and only contain the native structure in their core. Furthermore, having no tropocollagen as the basic building block for the design of new fibres is contained. Therefore, crosslinking between fibrils in the telopeptide region cannot occur, and reconstituted fibres from insoluble collagen lack biofunctionality, making their strength is reduced. Only acid-soluble collagen (ASC) and enzyme-treated collagen, mainly pepsin-solubilized collagen (PSC), retain the ability for self-assembly after extraction and are used in a bottom-up approach. Since the gelation and fibrillation of PSC require higher concentrations and telopeptides are cleaved [26,27], ASC is preferred for the spinning of collagen fibres for biomedical application. Fibres from ASC can be formed from tropocollagen molecules arranging in fibrils during fibrillogenesis, showing the characteristic D-banding of native collagen fibres over their complete length. But in this in vitro process, no natural crosslinking does occur, and therefore, reconstituted collagen fibres lack the mechanical strength, enzyme resistance, and thermal stability of their native counterpart [5,28]. Thus, the potential of collagen fibres for biomedical applications is limited [8,13,29]. To restore the mechanical properties and stability of fibre-based scaffolds and to enable textile processing, additional crosslinking steps are necessary. A big issue concerning biomedical application is the cytocompatibility of the crosslinked collagen. Introducing additional chemical and potentially reactive entities in the collagenous material often leads to acute inflammatory responses and fibrotic enclosure [30,31]. To prevent this foreign body response, cytocompatibility of the crosslinking method must be established.

In contrast to previous reviews on collagen crosslinking [32,33,34,35,36], the present work specifically focuses on the crosslinking of reconstituted collagen fibres from acid-soluble collagen. To date, only the study by Zeugolis et al. (2009) has systematically investigated the crosslinking of extruded collagen fibres [13]. In their work, the authors aimed to standardise protocols for collagen preparation, fibre fabrication, and testing to enable a direct comparability of 16 different crosslinking methods. The fibres were extruded from an atelocollagen solution, which is a form of collagen that has undergone enzymatic removal of N- and C-terminal telopeptides. As discussed previously, the removal of these telopeptide regions hinders natural self-assembly of tropocollagen into native fibrils and prevents the formation of the characteristic D-periodic banding pattern, which is essential for proper cell–matrix interaction and biological functionality [37,38]. Consequently, the resulting fibres might lack critical features of native collagens fibrillar structure, potentially compromising their cytocompatibility and mechanical integrity.

Nevertheless, the study by Zeugolis et al. demonstrated that “Chemical stabilisation through bifunctional agents or agents that promote matrix formation brought about more pronounced changes” compared to enzymatic or physical crosslinking methods [13]. This review provides a concise overview of collagen fibre crosslinking strategies, with an emphasis on selected chemical crosslinking methods. These are examined in greater detail with respect to their reaction mechanisms, effects on cytocompatibility, and influence on mechanical properties.

## 2. Crosslinking

Crosslinking is an established strategy for the targeted modification of biomaterials, which has been shown to enhance their degradability, as well as biological and mechanical properties, by inducing chemical and physical interactions between polymer chains [39]. In essence, there are three distinct methodologies for the crosslinking of polymers and proteins. The primary method under discussion is physical crosslinking. As is evident in the extant literature, physical crosslinking methods involve a variety of mechanisms, including, but not limited to, hydrogen bonding; complex formation; and hydrophobic, ionic, or electrostatic interactions [40]. The most common methods comprise the application of radiation (γ- and UV irradiation) and thermal treatment (mainly dehydrothermal treatment—DHT). DHT is defined as a combination of heat and vacuum conditions [41]. This process, referred to as dehydration, involves the removal of residual water from the collagen. Consequently, the presence of free carboxylic acid groups and amino groups leads to the formation of new amide bonds, thereby enhancing molecule stability [42,43]. The complete crosslinking of the collagenous matrix may require several days, and even after three days, the process may remain incomplete [43]. UV irradiation has been demonstrated to be a faster and more effective approach for collagen crosslinking [32]. This method results in the generation of free radicals within the aromatic side chains of tyrosine and phenylalanine, which subsequently react to form crosslinks [44]. However, both UV- and DHT-based crosslinking have been reported to result in relatively weak crosslinks and may cause partial denaturation of the collagen, i.e., a partial loss of the triple helical structure [43,45]. It is noteworthy that these methods do not introduce any chemical entities, thereby reducing the risk of inflammation and irritation.

In contrast, biological crosslinking employs an enzymatic approach to promote the introduction of covalent crosslinks between the protein molecules [46]. Enzymatic post-translational modifications have been demonstrated to play a crucial role in the in vivo crosslinking of collagen and elastin. Tropocollagen molecules that subsequently assemble into collagen fibrils are stabilised by covalent crosslinking catalysed by lysyl oxidases (LOXs) [47,48]. However, LOXs cannot easily be extracted or reproducibly expressed in bacterial systems [49]. Therefore, in vitro applications predominantly employ transglutaminases and peptidases as alternatives [32,34]. The primary benefits of enzymatic crosslinking are substrate specificity and the absence of cytotoxic by-products [50,51]. Conversely, enzymatic approaches are associated with several limitations, including high cost, limited scalability for industrial application, and potential structural alterations of the collagen matrix.

Conversely, chemical crosslinking offers greater versatility, particularly with regard to the controllability of the crosslinking degree, which directly correlates with enhanced thermal stability, mechanical strength, and resistance to enzymatic degradation. Hence, chemical crosslinking methods will be discussed in detail in the following section.

### 2.1. Chemical Crosslinking

Chemical crosslinking methodologies can be classified using various approaches, depending on the underlying chemical mechanisms involved. Mainly, crosslinkers are categorized into distinct groups based on their reactivity and mode of action. An overview is provided in Table 1. This review focuses on aldehydes, isocyanates, carbodiimides, epoxides, and some promising new concepts derived from plant extracts. Examples from these groups include glutaraldehyde (GA), the most commonly used collagen crosslinker; hexamethylene diisocyanate (HMDI); 1-ethyl-3-(3-dimethylaminopropyl) carbodiimide (EDC); and 1,4-butanediol diglycidyl ether (BDDE), which are more cytocompatible alternatives. Additionally, certain reagents and procedures to mitigate the cytotoxic effects of GA are presented.

**Table 1:** Overview of crosslinking reagents.

	Friess et al. [5]	Paul et al. [6]	Zeugolis et al. [13]	Ryglova et al. [52]	Adamiak et al. [32]
Tanning agents	x		x		
Aldehydes	x	x	x	x	x
Isocyanates (and Imidates)	x	x	x	x	
Carbodiimides	x	x	x	x	x
Acyl Azide	x		x		
Epoxides	x	x	x	x	
Carbohydrates		x	x		
Quinones		x	x		
NDGA (di-catechol)				x	
Carboxylic Acid Derivatives				x	
Polyphenols (Iridoid glycosides)			x	x	x
Chitosan					x

#### 2.1.1. Glutaraldehyde

**Figure 2:**
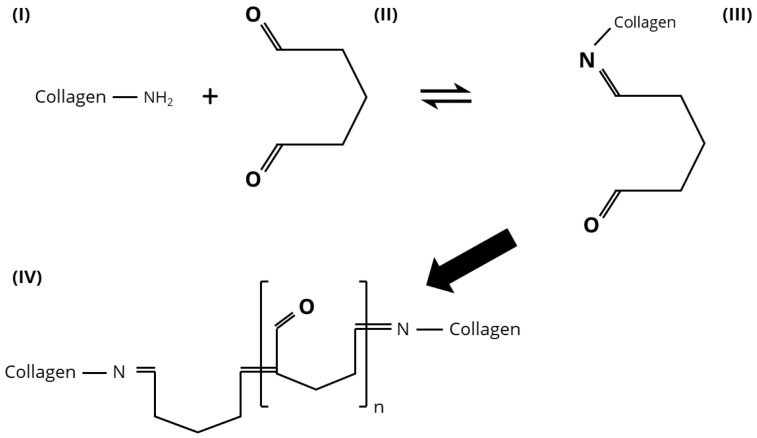
Basic crosslinking mechanism of Collagen with GA: Collagen (I) and GA (II) react, building a Schiff base intermediate (III). From there, the expected outcome is a GA polymer, building an imide-crosslinked collagen (IV).

Glutaraldehyde (GA) is the most commonly used chemical crosslinker for collagen due to its ability to significantly increase both chemical and enzymatic stability, leading to an improvement in mechanical properties [53,54]. However, the use of GA is associated with notable drawbacks, including cytotoxicity at elevated concentrations and the potential for post-implantation tissue calcification [35,55,56,57,58]. Furthermore, its low vapour pressure and strong odour complicate handling during the crosslinking procedure. GA functions as a homobifunctional crosslinking agent by reacting via its two aldehyde groups with functional groups on the target protein through various chemical mechanisms. In the case of collagen, crosslinking typically involves the reaction between the ε-amine groups of lysine or hydroxylysine residues and the aldehyde group of GA, initially forming Schiff base intermediates. These intermediates may subsequently undergo various rearrangement or condensation reactions, leading to a range of stable crosslinked structures, as detailed in references [32,59]. Figure 2 shows the basic crosslinking mechanism proposed by Olde-Damink et al. [59]. A key advantage of GA is its capacity to form intermolecular crosslinks by bridging the gap between adjacent collagen molecules. In contrast, most alternative crosslinkers are limited to intramolecular crosslinking because of their shorter molecular dimensions, which prevent them from spanning the gaps between collagen molecules.

Table 2 summarises the mechanical properties of collagen fibres crosslinked with glutaraldehyde, along with the underlying crosslinking methodologies. Two main approaches are evident: exposure to GA vapour and immersion in GA solution. Various exposure times and GA concentrations were employed, and the resulting mechanical properties of the collagen fibres in dry and wet states were examined. Due to the use of varying testing parameters, such as tensile strain rates of 10%, 25%, 33.3%, 50%, and 100% per minute, as well as differing soaking protocols for wet-state testing, direct comparison of the results is not feasible. Nonetheless, certain trends can be discerned. To improve understanding of the factors influencing GA crosslinking, some older work on reconstituted fibres from insoluble collagen dispersions is also included.

Kato et al. were the first to show that it is possible to fabricate strong reconstituted collagen fibres with wet strengths of 50 to 66 MPa using GA vapour for crosslinking, depending on the crosslinking duration and the strain rate during testing [60]. Interestingly, collagen fibres crosslinked for two days exhibited higher tensile strength than those crosslinked for four days. However, the differences are not statistically significant and were therefore not further addressed by the authors. The tensile strength of the GA crosslinked collagen fibres is comparable to that of native rat tail tendon fibres, which typically exhibit strengths of ca. 35 MPa. No data were reported for uncrosslinked reconstituted collagen fibres, limiting direct comparison. A moderate inflammatory response after implantation due to release of residual glutaraldehyde is mentioned. Building on this results, Law et al. reported the preservation of collagen for up to six months with a mild to moderate foreign body response using GA vapour [11]. In a follow-up study, Kato and Silver investigated continuously spun collagen fibres exposed to GA vapour for a shorter duration of one day, which resulted in reduced tensile strength and modulus [16]. These findings indicate that crosslinking degree and, consequently, the mechanical strength are dependent on exposure time.

Another influencing factor is the fibre diameter, as demonstrated by Dunn et al. [12]. Both factors seem to influence the penetration depth of the crosslinking reaction into the fibre. Longer exposure times allow for the formation of a higher number of crosslinks, progressively extending from the fibre surface into the core. In addition, a decrease in fibre diameter increases the surface-area-to-volume ratio, thereby enabling more rapid and uniform crosslinking across the entire fibre under the same conditions. Up to this point, insoluble collagen dispersions had served as the main source material for the in vitro production of collagen fibres.

Cavallaro et al. used acid-soluble collagen in a continuous spinning process and achieved mechanical properties comparable to those reported in previous studies using GA vapour crosslinking. The resulting fibres showed tensile strengths of 175 MPa in the dry state and 27.7 MPa in the wet condition [61]. Zeugolis et al. were the first to utilise an atelocollagen solution and, notably, introduced the immersion of collagen fibres in a 0.625% GA solution prepared in 0.01 M PBS overnight [13]. The resulting tensile strength and modulus in wet state—10.85 MPa and 6.9 MPa, respectively—were lower than those reported in earlier studies. This reduced mechanical performance may be attributed to the use of atelocollagen, which lacks the telopeptide regions essential for efficient fibril formation and subsequent intermolecular crosslinking [37,38]. In addition, the previously discussed factors, namely, crosslinker concentration, fibre diameter, and crosslinking duration, need to be considered. In the study by Zeugolis et al. [13], the GA concentration was lower, the exposure time shorter, and the collagen fibres had a larger diameter (260 µm dry, 306 µm wet), all of which might have negatively influenced the tensile properties, as outlined above. Siriwardane et al. used a 1% GA solution in H_2_O and soaked the fibres for 24 h [18]. The fibres, produced from insoluble collagen dispersion, had a significantly smaller diameter of 56 µm and showed strongly increased tensile strength results after crosslinking, reaching 136 MPa in the dry state. The corresponding uncrosslinked fibres had a strength of 59 MPa, indicating a 2.3-fold increase due to crosslinking. However, no data were provided for the mechanical performance under the wet condition, which would be relevant for evaluating their suitability for in vivo application.

Yaari et al. developed a wet spinning and drawing system aimed at decreasing the fibre diameter and increasing the mechanical properties by enhancing the alignment of the collagen molecules within the fibres [62]. They used GA for crosslinking directly in the wet spinning buffer (WSB) and crosslinked for 24 h at room temperature. This approach led to a further increase in tensile strength, reaching 151 MPa. However, these values might not be directly comparable to other studies because a recombinant human collagen was used as the base material.

Tonndorf et al. showed the effectiveness of GA crosslinking for acid-soluble collagen mono- and multifilament yarns to increase mechanical properties in the wet condition [10,19]. They used 1.0 wt.-% and 0.5 wt.-% GA dissolved in ethanol to achieve simultaneous crosslinking and dehydration during continuous fibre production. The resulting fibres exhibited strengths of 174 MPa (1.0 wt.-%) and 169 MPa (0.5 wt.-%) in the dry state and 66 MPa (1.0 wt.-%) and 40 MPa (0.5 wt.-%) in the wet state, respectively. The relatively modest tensile properties may be attributed to the short exposure time inherent to the continuous processing methods. Nevertheless, the fibres produced are well suited for further textile manufacturing and are comparable to the tensile strength of the human anterior cruciate ligament, which has a tensile strength of approximately 38 MPa [12,63]. Additionally, the integration of an online crosslinking strategy into the spinning process offers significant advantages for industrial scale-up, while also leading to notable improvement in tensile strength compared to non-crosslinked yarns. Still, the potential cytotoxic effects associated with GA remain a major concern. Several studies have demonstrated that GA can impair the cell proliferation-promoting properties of collagen due to its residual reactive aldehyde groups [35,55,56,57].

Targeting the cytotoxicity and calcification tendencies of GA-crosslinked materials, various approaches have been investigated to neutralise or eliminate these reactive residues. Matsuda et al. reported that sodium borohydride (NaBH_4_) and glycine can effectively react with free aldehyde groups and thus potentially reduce cytotoxicity [64]. In an effort to further improve collagen scaffold performance and reduce cytotoxic effects, Islam et al. [65] investigated a dual crosslinking strategy involving EDC and GA. This approach enhanced mechanical properties and enzymatic stability. Importantly, the cytotoxic effects typically associated with GA can be mitigated through post-treatment with sodium metabisulphite (SM) and sodium borohydride (SB), without significantly altering the scaffold’s mechanical integrity, enzymatic stability, or optical transparency. These findings suggest that neutralisation of residual aldehyde groups is a viable strategy for restoring cytocompatibility following GA-based crosslinking. Despite these approaches, the development of more cytocompatible alternatives remains imperative, and some promising candidates will be discussed in the following sections. Also, further research is essential to better understand and eliminate the cytotoxic effects associated with GA, thereby ensuring the safe application of crosslinked collagen-based materials in biomedical contexts.

#### 2.1.2. Hexamethylene Diisocyanate (HMDI)

An alternative crosslinking strategy that has been investigated early on is the use of isocyanates, such as hexamethylene diisocyanate. Similar to GA, HMDI reacts with free amine groups in lysine and hydroxylysine residues, enabling covalent crosslink formation [66]. As early as 1983, Chvapil et al. identified HDMI as a more cytocompatible crosslinker than GA for collagen sponges made from soluble collagen [67]. Upon subcutaneous implantation, these sponges demonstrated sustained cell infiltration and granulation tissue formation after 17 days with no observable toxic residues. Subsequent studies, however, reported a mild to moderate foreign body response and relatively rapid degradation of the material [66,68,69].

To date, the study by Zeugolis et al. remains the only comprehensive investigation on the crosslinking of reconstituted collagen fibres using HMDI [13]. Their findings indicate that HMDI is a promising crosslinker for reconstituted collagen fibres, especially under wet conditions, where up to 8% of dry-state tensile strength is retained. The mechanical properties of the crosslinked fibres are given in Table 3. However, despite its lower cytotoxic potential compared to GA, the translational potential of HMDI is still tempered by chemical safety: Residual HMDI is highly reactive and poses toxicity risks, including skin, respiratory, and immune sensitisation, if not thoroughly removed from the biomaterial. Furthermore, the use of HDMI is still associated with limited degradation resistance and a measurable foreign body response. Therefore, in the context of cytocompatibility and long-term biomedical applications, further alternatives need to be explored and evaluated.

#### 2.1.3. 1-Ethyl-3-(3ʹ-Dimethyl Amino Propyl) Carbodiimide (EDC)

A commonly used ’zero-length crosslinker’ in biomedical research is 1-ethyl-3-(3-dimethylaminopropyl) carbodiimide hydrochloride (EDC). Unlike conventional crosslinkers, EDC does not introduce additional chemical moieties into the final crosslinking structure, as the reaction results in direct covalent bonding between functional groups of the target molecules. Moreover, EDC does not contain inherently cytotoxic residues and is rendered inactive upon completion of the crosslinking reaction, eliminating concerns related to residual reactivity or toxicity [70,71]. Due to its favourable cytocompatibility profile, lack of discolouration, and absence of cytotoxic by-products, EDC has been widely employed in the crosslinking of collagen and other biopolymers, particularly in applications where the preservation of biological function is critical [13,72,73,74].

**Figure 3:**
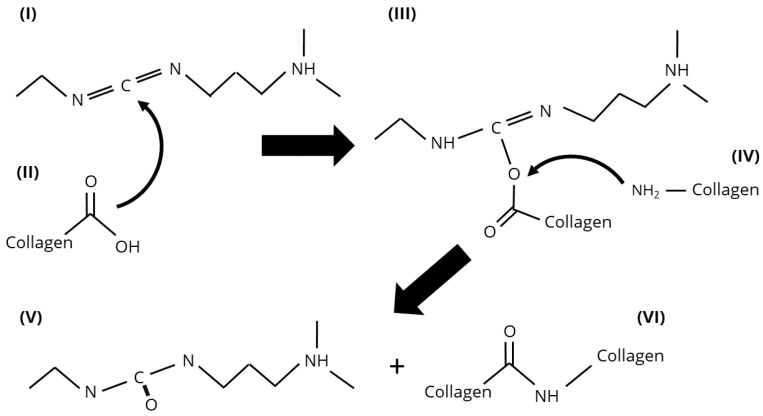
Reaction mechanism of EDC crosslinking of collagen.

The crosslinking mechanism of EDC involves the activation of carboxyl groups, resulting in the transient formation of an O-acyl isourea intermediate. Subsequent nucleophilic attack by a primary amine leads to the formation of a stable amide bond, effectively linking the original carboxyl and amine groups without leaving a residual spacer molecule—hence the term zero-length crosslinker [75,76]. A schematic representation of this reaction mechanism is provided in Figure 3.

Although various strategies for the crosslinking of collagen fibres have been investigated in the literature, the specific mechanics and biological effects of EDC-mediated crosslinking in insoluble collagen fibres remain insufficiently understood. While EDC is a commonly used carbodiimide-based crosslinker, its interaction with structurally mature collagen systems, such as reconstituted or native-like fibres, requires further systematic investigation. Kew et al. demonstrated the efficacy of EDC/NHS-mediated crosslinking (25 mM EDC and 12.5 mM NHS) in stabilising synthetic collagen fascicles, which supported tendon fibroblast adhesion. The fibroblasts exhibited a phenotypical flattened morphology, indicative of good cytocompatibility and substrate interaction [77]. In a related study, Ahmad et al. investigated the effect of EDC concentrations on the biological characteristics of crosslinked collagen fibres [78]. Owing to the water solubility of the EDC and the formation of non-toxic urea as the only by-product, EDC crosslinking is generally considered to be cytocompatible. Ahmad et al. observed that increasing EDC concentration enhanced the fibres’ resistance to enzymatic degradation, specifically collagenase-mediated breakdown. However, this stabilisation was accompanied by a decline in cell adhesion and proliferation. At higher EDC concentrations (25 mM), cells appeared more rounded and less spread, whereas at lower concentrations (0.25 mM), cells exhibited a more elongated and flattened morphology—suggesting improved cell–matrix interaction at reduced crosslinking densities. The authors therefore suggested that lower crosslinker concentrations may offer a more beneficial balance between degradability, mechanical properties, and biological response. However, an optimal concentration and molar ratio of EDC relative to available carboxylic acid groups has yet to be determined.

Table 4 provides an overview of the crosslinker quantities, methodology, and resulting mechanical properties. The swelling behaviour and the ratio of wet to dry tensile strength are given in Table 5 and are compared to GA-crosslinked fibres. As shows, EDC-crosslinked fibres demonstrate significantly lower improvement in mechanical strength and greater swelling compared to GA crosslinked fibres. Specifically, EDC-treated fibres swell at least twice as much as their GA-crosslinked counterparts, which is a trend also confirmed by comparative data from Yaari et al. [62]. Nonetheless, EDC remains a promising candidate for biomedical applications due to its superior cytocompatibility and sufficient improvement in mechanical and proteolytic (enzymatic) resistance. Despite its lower reinforcement potential, EDC-treated fibres show improved cell migration rates compared to physically crosslinked fibres [79]. Critically, EDC does not cause significant calcification or cytotoxicity when thoroughly washed, reducing risks compared to crosslinkers like glutaraldehyde. Additionally, the ability to immobilise bioactive molecules (e.g., heparin, growth factors) further supports its clinical utility for engineered tissues, particularly in cardiovascular and regenerative therapies [80]. The primary translational limitations are potential effects on integrin cell signalling and the need for careful process optimisation to balance mechanical strength with cellular compatibility [81].

Cavallaro et al. recognised the potential of EDC as a crosslinking agent for collagen fibres, braids, and knitted fabrics as early as 1994 [61]. As one of the few studies involving acid-soluble collagen fibres produced via a continuous spinning process, it warrants closer examination. In this study, collagen fibre-based textile structures were produced and evaluated as potential tissue-engineered implants for anterior cruciate ligament replacement in a dog model. The effects of EDC crosslinking were compared to those of non-crosslinked and GA-crosslinked fabrics. Both crosslinking procedures led to an increased shrinkage temperature, which is an indicator of triple helix stabilisation in collagen, and increased tensile strength in the wet state. For EDC-treated samples, both parameters improved with increasing crosslinking times and EDC concentrations. Notably, stabilisation of the triple helix occurred earlier than the increase in tensile strength, which the authors attributed to the more rapid formation of intramolecular crosslinks compared to slower-forming intermolecular bonds. The fabrication of collagen braids and tricot fabrics further demonstrates the textile processability of the collagen fibres. In addition to sufficient tensile strength, the fibres exhibited adequate flexibility for textile manufacturing, which was achieved by rinsing the threads in water, likely causing partial rehydration that facilitated fibril mobility. No signs of adverse tissue response were observed upon implantation, and normal wound healing occurred. The implants maintained their structural integrity and functional performance throughout the remodelling period. Gentleman et al. confirmed that EDC crosslinking improves the mechanical properties of the fibres in the swollen state [82]. However, decreasing elastic modulus and tensile strength were reported with increasing fibre diameter. This effect was attributed to a higher probability of structural defects, such as the misalignment of collagen substructures, and to a reduced surface area-to-volume ratio. As a result, surface-initiated crosslinking is unable to sufficiently penetrate thicker fibres within the same reaction time and crosslinker concentration, leading to incomplete crosslinking across the fibre cross-section. These findings corroborate earlier reports [12,86,87].

Caruso et al. reported enhanced crosslinking efficiency of collagen fibres using EDC when the reaction was conducted in an acetone bath rather than in aqueous media [83]. The acetone environment not only prevents rapid hydrolysis of EDC but also induces fibre dehydration [70,83,88], which is a finding corroborated by Bou-Akl et al. [14]. In their study, crosslinking with 10 mM EDC increased the tensile strength of collagen fibres from 0.82 MPa to 7.8 MPa. When acetone was used as the solvent instead of water tensile strength further increased to 39.1 MPa. This improvement was attributed to enhanced proximity of amino acid residues involved in crosslinking, facilitating bond formation and allowing shorter EDC molecules to form effective intermolecular crosslinks [70]. Furthermore, the acetone environment inhibits the disruption of hydrogen and electrostatic bonds that would otherwise occur through water molecule infiltration between collagen chains [42].

Several studies have explored the addition of N-hydroxy succinimide (NHS) to support EDC-mediated crosslinking [13,14,77]. NHS creates amine-reactive NHS ester intermediates, which are more resistant to hydrolysis than the transient O-acylisourea intermediates typically formed during EDC crosslinking, while still enabling effective amide bond formation at physiological pH levels. Bou-Akl et al. compared fibres crosslinked in aqueous or acetone-based EDC solutions with those treated using EDC/NHS in water [14]. While the addition of NHS resulted in lower dry-state tensile strength (122.9 MPa vs. 189.2 MPa), the EDC/NHS-treated fibres exhibited improved strength retention under wet conditions (10.5 MPa vs. 7.8 MPa). However, acetone-based crosslinking proved superior in enhancing tensile strength in both dry and wet conditions, making it a preferred approach over NHS supplementation in this context.

In another study, Enea et al. demonstrated that combining EDC with epoxy crosslinker, specifically ethylene glycol diglycidyl ether (EGDE), can produce a favourable balance between cytocompatibility, mechanical strength, and enzymatic resistance [84]. The enhanced crosslinking density achieved by exploiting the multiple nucleophilic sites along the EGDE backbone contributed to improved scaffold durability. However, cell adhesion on these dual-crosslinked materials was reduced, likely due to the presence of residual EDGE, which may be more cytotoxic than EDC alone. A subsequent in vivo study in an ovine model confirmed the superior cytocompatibility of EDC-only crosslinking compared to the combination of EDC and EGDE [89]. A more recent study from 2021 by Dasgupta et al. also showed an increase in tensile strength from 6.1 ± 1.2 for an untreated collagen fibre sample to 16.6 ± 1.5 for an EDC-treated sample in the wet state [85]. Wet-extruded fibres from collagen type I with intact telopeptides (telocollagen) were immersed in 0.25 mM EDC in a 70% EtOH solution for 24 h to achieve crosslinking. Unfortunately, no data are given regarding cytoxicity. The comparatively low tensile strength might be mainly due to the low amount of crosslinker used. To date, relatively few studies have investigated the cytocompatibility of fibre-based collagen constructs. Nevertheless, successful in vitro and in vivo outcomes have been reported. Cavallaro et al. and Bou-Akl et al. demonstrated good cellular attachment and viability of bone marrow-derived mesenchymal stem cells on fibres crosslinked with 10 mM EDC, supporting their potential use in regenerative medicine applications [14,61]. Kew et al. demonstrated the effectiveness of EDC/NHS crosslinking (25 mM EDC and 12.5 mM NHS) in promoting tendon fibroblast attachment on synthetic collagen fascicles, with the fibroblasts exhibiting a flattened morphology indicative of favourable cell-material interaction [77]. Ahmad et al. further investigated the effect of EDC concentrations on the biological properties of crosslinked collagen fibres [78]. Owing to the water solubility of EDC and non-toxic nature of its by-product, urea, EDC is generally regarded as cytocompatible. However, Ahmad et al. observed that increasing the EDC concentration enhanced resistance to collagenase-mediated degradation but concurrently reduced cell adhesion and proliferation. At a higher concentration (25 mM EDC), fewer cells with a rounded morphology were present on the fibre, while at a lower concentration (0.25 mM), cells exhibited a more spread and flattened phenotype, suggesting improved cell–matrix interactions. The authors therefore suggested that reduced crosslinker concentrations may offer a favourable balance between enzymatic stability, mechanical reinforcement, and biological response. However, an optimal EDC concentration and a defined molar ratio relative to the available carboxylic acid groups have yet to be established.

#### 2.1.4. 1,4-Butanediol Diglycidyl Ether (BDDE)

**Figure 4:**
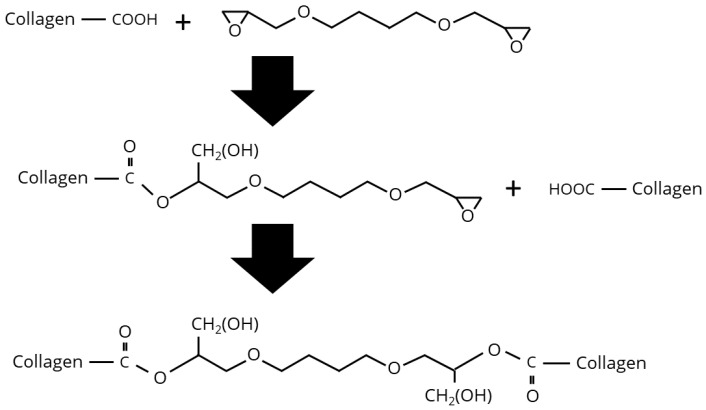
Reaction of the BDDE epoxide groups with the carboxyl residues of glutamic and aspartic acid, forming ester bonds.

**Figure 5:**
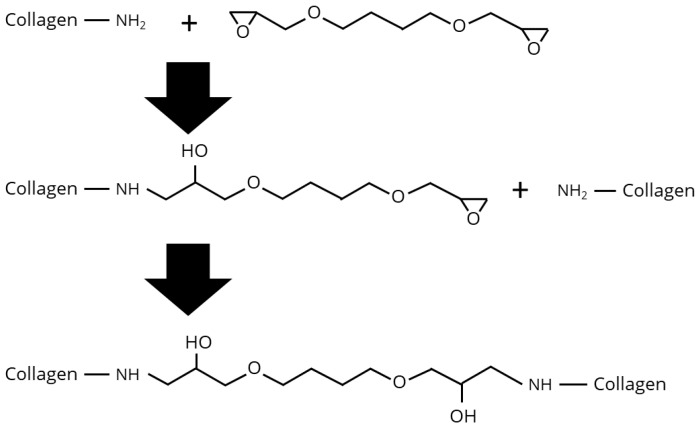
Reaction of the BDDE epoxide groups with the amine groups of hydroxylysine residues, forming secondary amines.

1,4-Butanediol diglycidyl ether (BDDE) is a well-established crosslinker commonly used for hyaluronic acid in cosmetic applications. Beyond this, BDDE has identified as a suitable crosslinker for electrospun collagen nanofibres, with its cytocompatibility and ability to support cell adhesion [90,91,92]. Moreover, BDDE crosslinking has been shown to improve the physicochemical properties of collagen, and the influence of parameters such as BDDE concentration, reaction time, temperature, and pH on the crosslinking efficiency has been investigated [93,94]. Despite its extensive application in collagen films and hydrogels, no published data are currently available regarding the crosslinking of collagen fibres using BDDE. Chemically, BDDE is a homobifunctional epoxide compound capable of reacting with amine, hydroxyl, or sulfhydryl groups, leading to the formation of secondary amine, ether, or thioether bonds, respectively. According to Zeeman et al., under acidic conditions (pH 4–6) BDDE’s epoxide groups react with the carboxyl side chains of glutamic and aspartic acid residues, resulting in the formation of ester bonds [75,93]. In contrast, under basic conditions (pH 8–10), the epoxide groups preferably react with the amine groups of hydroxylysine residues in collagen, forming secondary amine linkages (see Figure 4 and Figure 5).

Koh et al. investigated the use of BDDE to crosslink collagen-based corneal substitutes derived from type I porcine collagen [95]. They observed improvements in both elasticity and tensile strength, as well as enhanced proliferation of corneal epithelial cells. In accordance with Zeeman et al., they identified the primary crosslinking mechanism under basic condition to be the epoxide ring opening reaction with amine groups, forming stable secondary amines. Under acidic conditions, the reaction pathway shifts toward esterification, which was found to be insufficient for the formation of stable collagen hydrogels. To overcome this limitation at pH 5, Koh et al. employed Cu(II) tetrafluoroborate (Cu(BF_4_)_2_·xH_2_O) as a catalyst to facilitate secondary amine bond formation via epoxide ring opening, as a strategy previously validated in the literature [96]. A key drawback of BDDE crosslinking lies in its considerably slow reaction kinetics compared to carbodiimide-based systems such as EDC. While this slower rate may enable greater control over the crosslinking process, it may be disadvantageous in continuous fibre spinning process applications where rapid crosslinking is desired. Notably, Koh et al. demonstrated that a combined crosslinking approach using BDDE and EDC/NHS at pH 5 can lead to synergistic improvements in crosslinking efficiency [95]. These hydrogels made by sequential BDDGE and EDC/NHS crosslinking at pH 5 achieved gelation within 72 h at room temperature, with optimal conditions at a 1:1 BDDGE–collagen-NH_2_ ratio and 30% Cu catalyst.

BDDE-crosslinked collagen is noted for its reduced cytotoxicity compared to agents like glutaraldehyde, leading to better biocompatibility and cellular performance, including improved adhesion, proliferation, and migration when used in skin, wound healing, and cartilage regeneration products. The relatively slow crosslinking kinetics of BDDE allow for homogenisation and the incorporation of bioactive agents, supporting custom formulations for cell and drug delivery. BDDE is also already widely used as a crosslinking agent in commercially available dermal fillers and hydrogels for soft tissue augmentation, with an excellent safety profile in clinical practice [97]. It offers improved mechanical stability, tunable degradation, and safety if unreacted BDDE is completely removed to prevent possible adverse effects. Overall, its regulatory approval in several commercial filler products underscores BDDE’s broad translational utility for collagen-based biomaterials.

#### 2.1.5. Divinyl Sulfone (DVS)

Another highly reactive crosslinking agent known from crosslinking applications with hyaluronic acid is divinyl sulfone [98,99]. It reacts via two electron-deficient vinyl groups to form covalent bonds with the nucleophilic functional groups, mainly the amino or hydroxyl groups of collagen, HA, and other proteins to create a chemically stabilized, three-dimensional network. To date, there are no studies on the DVS crosslinking of collagen fibres. Nevertheless, divinyl sulfone was shown to offer advantages such as methodological simplicity and high cytocompatibility [100,101], offering potential for tailored physical and biological performance in regenerative medicine and bioengineering applications by enabling the fine-tuning of scaffold properties. This underscores the potential of DVS as a crosslinker for nanostructured collagen systems like collagen-based nanogels, nanofibres, and nanocomposites, as well as wet-spun collagen fibres, and should be considered in future research.

#### 2.1.6. Natural Crosslinkers Derived from Plant Extracts

An alternative strategy to mitigate cytotoxic effects and improve the cytocompatibility of collagen-based biomaterials involves the use of natural crosslinkers derived from plant extracts. A recent review article discusses a range of such crosslinkers with potential application in tissue engineering [40]. These crosslinkers generally offer high cytocompatibility, low toxicity, and broad availability while often providing additional bioactive properties, including anti-microbial, anti-inflammatory, and antioxidant effects. However, natural crosslinkers are typically associated with higher costs and lower crosslinking efficiency compared to synthetic alternatives.

One of the most widely studied natural crosslinkers is genipin (Gp), a bioactive compound extract from the fruits of the *Genipa americana* or *Gardenia jasminoides Ellis*. Recognised as safe for food application, genipin is commonly used in combination with chitosan in tissue engineering and alternative medicine due to its antimicrobial and anti-inflammatory properties [102,103,104,105]. Genipin can react with the primary amine groups of collagen, forming amide bonds [106], and has been shown to preserve the fibrous structure of electrospun collagen fibres in aqueous environments [107]. Different crosslinking conditions have been investigated, with optimal results reported for the genipin concentration of 0.03 M in ethanol or isopropanol containing 3–5% water. Under these conditions, primary human fibroblasts successfully adhered to all genipin-crosslinked samples. Notably, all fibre samples developed a deep blue colouration upon hydration, which is a characteristic feature of genipin crosslinking. Siriwardane et al. crosslinked collagen fibres from a mixture of soluble and insoluble collagen type I [18]. The fibres showed a strongly increased modulus of 2394 ± 148 MPa, as well as an increased cell survival rate of dorsal root ganglion (DRG) neurons and Schwann cells compared to the GA positive control. Furthermore, in a recent study, Lin et al. showed the potential of genipin crosslinking on electrochemical aligned collagen threads (ELACs) [108]. ELAC threads crosslinked with 0.1% and 2% genipin exhibited bundled fibres, with denser packing observed at 2%. Furthermore, an extended incubation period with 0.1% genipin led to a substantial augmentation in fibre packing. Also, an increase in crosslinker concentration from 0.1% to 2.0% has been shown to elicit an increase in Young’s modulus from 1.51 MPa to 2.87 MPa and an increased crosslinking degree in a TNBS assay.

Quercetin, another natural occurring crosslinker, is a flavonoid commonly found in grapes, berries, and tomatoes. Zhai et al. demonstrated that quercetin crosslinking of a porcine heart valve ECM significantly increased tensile strength and denaturation temperature [109]. The treated ECM retained its elasticity and tensile strength after storage in D-Hanks solution for 30 days, and a vascular endothelial cell exhibited sustained proliferation without signs of calcification in a simulated body fluid. Depending on the concentration used, the crosslinked ECM exhibited a yellow to brown colour change.

In 2010, Zeugolis et al. investigated the use of Myrica rubra extract as a crosslinker for extruded collagen fibres [110]. The resulting fibres displayed a higher denaturation temperature than those crosslinked with GA. Additionally, the fibre diameter and swelling ratio decreased, while the tensile strength and modulus increased. As with other plant-derived agents, a brown discolouration of the fibres was observed.

A comparison of some plant extract crosslinked collagen fibres is given in Table 6.

## 3. Conclusions and Future Perspectives

There are quite a few successful examples of collagen-based products in clinical application, mainly focusing on wound dressings, skin substitutes, and dermal fillers [97]. However, to this date, there are no textile collagen based products because the consistent, large-scale production of clinical-grade collagen fibres suitable for the manufacture of medical devices has remained a challenge [13,84,111]. Recently, a collagen–UHMWPE co-braid suture was developed with mechanical properties that meet the high demands of orthopaedic procedures [112]. But benchtop, animal, and clinical studies are still pending. Still, this example shows the high potential of strong, cytocompatible collagen fibres. To enhance the mechanical and enzymatic resistance of collagen fibres for the production of textile scaffolds for tissue engineering, fibre-based wound dressings or similar biomedical products, post-fabrication crosslinking remains an essential processing step. Glutaraldehyde (GA), although widely used due to its ability to significantly increase mechanical strength and thermal stability, as well as its resistance to enzymatic degradation, is associated with notable cytotoxicity, as well as the induction of foreign body response and tissue calcification. These adverse effects underscore the need for alternative crosslinking strategies that provide effective stabilisation without compromising cytocompatibility.

In response, various synthetic and natural crosslinking agents have been investigated as a potential substitute for GA. Among the synthetic alternatives, hexamethylene diisocyanate (HMDI), 1-ethyl-3-(3-dimethylaminopropyl) carbodiimide (EDC), 1,4-butanediol diglycidyl ether (BDDE), and divinyl sulfone (DVS) have demonstrated the ability to crosslink collagen effectively while exhibiting reduced cytotoxicity. These reagents offer improved cytocompatibility and potential for clinical translations, although their capacity to elicit immune or foreign body responses—particularly at higher concentrations—remains a concern. An overview of several studies on the inflammatory response in vitro and host response of in vivo models of collagen-based devices is given in ref. [111].

Conversely, natural crosslinkers derived from plant-based sources (e.g., genipin, quercetin, Myrica rubra extract) offer distinct advantages in terms of biological safety, biodegradability, and the potential to confer bioactive effects, including antimicrobial, antioxidant, and anti-inflammatory properties. However, these agents are often associated with higher production costs and lower crosslinking efficiency compared to synthetic crosslinkers. Despite these limitations, plant-derived extracts have demonstrated the potential to crosslink collagen fibres, thereby improving their thermal and mechanical stability in a more cytocompatible manner than conventional synthetic agents such as formaldehyde, glutaraldehyde, and isocyanates, provided that discolouration of the fibre or the final product is not a critical limitation.

Table 7 gives a summary and comparison of the aforementioned collagen crosslinking strategies in terms of cost, time, outputs, and safety.

As described in more detail in Section 2.1.1 and Section 2.1.3, studies by Islam et al. [65] combining GA and EDC crosslinking, as well as Enea et al. [84] combining EDC with an epoxy compound, show that there is an enormous potential of dual crosslinking strategies to integrate the complementary strengths of different agents. For example, combining EDC with BDDE, a more cytocompatible epoxy compound than EGDE, could offer an optimized balance between mechanical performance and biological safety. However, further investigation is necessary to evaluate such combinations, particularly with respect to cytotoxicity, residual reagent clearance, and cell–matrix interactions.

Finally, while in vitro studies provide valuable insight into cytocompatibility and mechanical behaviour, in vivo validation remains essential. Cellular responses observed in vitro may not translate directly to complex physiological environments and do not account for potential inflammatory, fibrotic, or immune-mediated reactions. As such, comprehensive in vivo studies are critical to assess the long-term cytocompatibility, degradability, and functional integration of crosslinked collagen scaffolds. Future research should focus on the following:

- Optimising crosslinking conditions (e.g., pH, solvent systems, catalyst use) to balance crosslinking efficiency with cytocompatibility.
- Elucidating long-term in vivo responses to various crosslinkers, particularly regarding immune modulation, biodegradability, and calcification potential.
- Exploring hybrid crosslinking strategies, such as combinations of synthetic and natural agents, to synergistically harness their respective advantages.
- Developing scalable and fibre-compatible crosslinking methods, particularly for continuous manufacturing processes such as electrospinning or wet spinning.

Overall, the selection of an appropriate crosslinking method should be tailored to the specific application requirements, considering both material performance and biological safety.

## Figures and Tables

**Table 2:** Overview of the mechanical properties of collagen fibres crosslinked with glutaraldehyde. (n.a. not available).

Ref.	Year	Crosslinking Procedure	Dry/Wet	Young’s Modulus [MPa]	Strength [MPa]	Strain at Break [%]	Maximum Force [N]
[60]	1989	25% (*w*/*v*) GA vapour; 2 or 4 d	dry (2 d)	4070 ± 401	152 ± 43.6	14.8 ± 1.8	n.a.
			wet (2 d)	503 ± 127.7	59.2 ± 17.9	13.8 ± 2.9	n.a.
			dry (4 d)	3550 ± 311	142.4 ± 38	11.8 ± 4.7	n.a.
			wet (4 d)	403 ± 80.9	55.5 ± 11.8	14.4 ± 3.6	n.a.
[11]	1989	25% (*w*/*v*) GA vapour; 4 d	dry	3653 ± 547	176.6 ± 29.1	17.2 ± 4.61	0.313 ± 0.065
			wet	482.1 ± 93.4	42.9 ± 9.55	16.2 ± 2.1	0.142 ± 0.034
[16]	1990	25% (*w*/*v*) GA vapour; 1 d	dry	n.a.	n.a.	n.a.	n.a.
			wet	270 ± 69.3	36.9 ± 7.9	16.6 ± 3.04	n.a.
[12]	1993	25% (*w*/*v*) GA vapour; 1 d	dry_280	n.a.	175	n.a.	0.06
			dry_580	n.a.	205	n.a.	0.43
			dry_860	n.a.	80	n.a.	0.38
			wet_280	n.a.	110	n.a.	0.05
			wet_580	n.a.	55	n.a.	0.25
			wet_860	n.a.	25	n.a.	0.18
[61]	1994	GA vapour overnight	dry	n.a.	175 ± 19	n.a.	n.a.
			wet	n.a.	27.7 ± 3.1	n.a.	n.a.
[13]	2009	0.625% GA in 0.01 M PBS	dry	34.2 ± 19.1	31.89 ± 9.93	55 ± 5	1.62 ± 0.23
			wet	6.9 ± 3.29	10.85 ± 2.85	43 ± 4	0.77 ± 0.04
[17]	2010	25% (*w*/*v*) GA vapour; 18–24 h	wet	n.a.	93.9 ± 19.2	n.a.	n.a.
[18]	2014	1.0% (*v*/*v*) GA in H_2_O; 24 h	Coll_dry	707 ± 68	59 ± 18	10.9 ± 1.6	n.a.
			GA_dry	2821 ± 168	136 ± 2.6	10.8 ± 1.9	n.a.
[62]	2016	0.1% GA in WSB	wet	888 ± 153	151 ± 31	20.5 ± 1.95	n.a.
[10]	2018	25% (*w*/*v*) GA solution in 96% EtOH	Coll_dry	2590 ± 336	187.6 ± 29.4	30.3 ± 2.6	n.a.
			Coll_wet	n.a.	n.a.	n.a.	n.a.
			GA_dry	2576 ± 252	173.6 ± 8.4	25.5 ± 3.8	n.a.
			GA_wet	854 ± 266	65.8 ± 2.8	14.1 ± 2.9	n.a.
[19]	2020	25% (*w*/*v*) GA in 96% EtOH	dry	3534 ± 399	169 ± 11	n.a.	n.a.
			wet	281 ± 15	40.4 ± 4.4	n.a.	n.a.

**Table 3:** Overview of the mechanical properties of collagen fibres crosslinked with hexamethylene diisocyanate.

Ref.	Year	Crosslinking Procedure	Dry/Wet	Young’s Modulus [MPa]	Strength [MPa]	Strain at Break [%]	Maximum Force [N]
[13]	2009	5% HMDI sol. in 100% 2-propanol	dry	14.79 ± 5.78	20.05 ± 8.46	29 ± 11	1.53 ± 0.10
			wet	4.39 ± 2.13	17.25 ± 5.92	45 ± 15	1.11 ± 0.41

**Table 4:** Overview of the mechanical properties of collagen fibres crosslinked using EDC.

Ref.	Year	Crosslinking Solution (Duration)	Type	Young’s Modulus [MPa]	Strength [MPa]	Strain at Break [%]
[61]	1994	50 mM EDC in 90% acetone (8 h)	Coll_dry	n.a.	224 ± 19	n.a.
			Coll_wet	n.a.	1.2 ± 0.2	n.a.
			EDC_dry	n.a.	197 ± 18	n.a.
			EDC_wet	n.a.	23.9 ± 2.7	n.a.
[82]	2003	1% (*w*/*v*) EDC in H_2_O (24 h)	wet_510	484.7 ± 76.3	50 ± 13.4	n.a.
			wet_1020	359.6 ± 28.4	36 ± 5.4	n.a.
			wet_1270	269.7 ± 11.9	24.7 ± 2.9	n.a.
[83]	2004	EDC in 90% acetone (24 h)	wet_6 mM	n.a.	28.0	n.a.
			wet_10 mM	n.a.	43.0	n.a.
			wet_25 mM	n.a.	31.0	n.a.
[79]	2007	1% (*w*/*v*) EDC in H_2_O (24 h)	Coll_wet	4.0 ± 1.2	1.5 ± 0.2	42 ± 12
			EDC_wet	68 ± 31	11 ± 4	17 ± 4
[13]	2009	1.731/0.415 g EDC/NHS (EN) in 0.5 M MES (overnight)	Coll_dry	154.23 ± 22.09	111.85 ± 10.11	37 ± 7
			Coll_wet	3.78 ± 1.1	2.97 ± 0.87	33 ± 7
			EDC_dry	59.18 ± 25.2	125.89 ± 39.21	53 ± 8
			EDC_wet	1.76 ± 0.33	3.16 ± 0.63	54 ± 11
[84]	2011	1:2:0.5 C/EDC/NHS in 0.01 M MES (1 h)	EN (wet)	19.3 ± 1.7	4.6 ± 0.4	23.2 ± 2.0
			EN/EGDE (wet)	46.2 ± 4.9	10.5 ± 1.3	23.1 ± 2.3
[77]	2012	25/12.5 mM EN in acetone/PBS (2 h)	EN (wet)	183.5 ± 76.2	25.1 ± 9.2	13.5 ± 4.4
			EN/EGDE (wet)	276.8 ± 168.4	49 ± 22.1	17.3 ± 5.6
[14]	2013	10 mM EDC in H_2_O or 90% acetone (24 h)	Coll_dry	2340 ± 630	151.2 ± 83.5	11 ± 8
			Coll_wet	4.7 ± 1.1	0.82 ± 0.1	31 ± 12
			EDC_dry	2582 ± 410	189.2 ± 102.3	14 ± 10
			EDC_wet	77 ± 6	7.8 ± 1	14 ± 4
			EDC_Ac_dry	5000 ± 510	408 ± 39.5	18 ± 3
			EDC_Ac_wet	474.7 ± 110	39.1 ± 6.6	12 ± 3
[85]	2021	0.25 mM EDC in 70% EtOH (24 h)	EDC (wet)	∼480 ± 240	16.6 ± 1.5	∼5 ± 1
		0.25/0.125 mM EDC/NHS in 70% EtOH (24 h)	EN (wet)	∼240 ± 100	30.2 ± 1.0	∼19 ± 7

**Table 5:** Percentage of swelling calculated from dry and wet fibre diameters and proportion of wet strength to dry strength.

Ref.	Type	Swelling	Fraction Wet Strength/Dry Strength
[14]	uncrosslinked	282.92%	0.54%
	EDC crosslinked	168.25%	4.12%
	EDC crosslinked + acetone	122.18%	9.58%
[61]	uncrosslinked	-	0.54%
	EDC crosslinked	-	12.13%
[13]	EDC crosslinked	123.35%	2.51%
	GA crosslinked	17.69%	34.02%
[16]	GA crosslinked	23.77%	-
[12]	GA crosslinked (280)	25.00%	62.86%
	GA crosslinked (580)	41.51%	26.83%
	GA crosslinked (860)	21.79%	31.25%
[10]	GA crosslinked	-	37.90%

**Table 6:** Overview of the mechanical properties of collagen fibres crosslinked with plant extracts.

Ref.	Year	Crosslinking Procedure	Dry/Wet	Young’s Modulus [MPa]	Strength [MPa]	Strain at Break [%]
[110]	2010	1% M. rubra aq. overnight	control_wet	3.78 ± 1.10	2.97 ± 0.87	33 ± 7
			MR_wet	23.10 ± 9.45	28.18 ± 8.51	15 ± 4
[18]	2014	1% (*v*/*v*) Gp aq. 24 h	Gp_dry	2394 ± 148	222 ± 74	16.4 ± 1.3
			Coll_dry	707 ± 68	59 ± 18	10.9 ± 1.6
[108]	2024	10 mL of 0.1% (*w*/*v*) Gp solution in 90% EtOH; 4 h	wet	1.51 ± 0.74	∼ 0.25	∼ 18
		10 mL of 2% (*w*/*v*) Gp solution in 90% EtOH; 4 h	wet	2.87 ± 1.59	∼ 0.65	∼ 16

**Table 7:** Comparison of the aforementioned crosslinkers and strategies for the crosslinking of collagen fibres.

Crosslinker	Cost	Time	Mechanical Output	Safety	Cytocompatibility	Translational Potential	Citations
EDC	Moderate	Fast	High stiffness	High	Moderate	Widely used, needs balance	[13,34,39,59,70,72,74,78,85]
BDDE	High	Moderate	Robust	Good	Good	Good for engineered tissues	[90,91,92,93,94]
DVS	Moderate	Fast	Strong, stable network	Acceptable	Variable	Limited, industrial focus	[98,99,101]
Glutaraldehyde	Low	Fast	Very strong, denaturing	Low	Low (cytotoxic)	Limited for implants	[11,12,13,14,16]
Genipin	Moderate	Slow	Good, cell-favorable	Very high	High	High, natural crosslinker	[18,34,108]
Quercetin	Low	Slow	Moderate, antioxidant	High	High	High, natural crosslinker	[109]
Myrica rubra	Low	Slow	Moderate, antioxidant	High	High	High, natural crosslinker	[110]
HMDI	Moderate	Fast	Strong, possible flexibility	Low	Low to moderate	Limited, needs purification	[66,67,69]

## Data Availability

No new data were created.

## Abbreviations

The following abbreviations are used in this manuscript:

ASC	Acid-solubilised collagen
BDDE	1,4-butanediol diglycidyl ether
DHT	Dehydrothermal treatment
DVS	Divinyl sulfone
ECM	Extracellular matrix
EDC	1-ethyl-3-(3ʹ-dimethyl amino propyl) carbodiimide
EGDE	Ethylene glycol diglycidyl ether
ELACs	Electrochemical aligned collagen threads
EN	EDC/NHS
GA	Glutaraldehyde
Gp	Genipin
HMDI	Hexamethylene diisocyanat
n.a.	not available
NHS	N-hydroxy succinimide
PSC	Pepsin-solubilised collagen
SM	Sodium metabisulphite
SB	Sodium borohydride
WSB	Wet-spinning buffer
